# Effects of the Addition of Microbial Agents After Dazomet Fumigation on the Microbial Community Structure in Soils with Continuous Cropping of Strawberry (*Fragaria × Ananassa* Duch.)

**DOI:** 10.3390/microorganisms13061178

**Published:** 2025-05-22

**Authors:** Ran Wu, Yan Li, Jian Meng, Jiangwei Han

**Affiliations:** 1 Shijiazhuang Academy of Agricultural and Forestry Sciences, Shijiazhuang 050041, China; candy58906@163.com (R.W.); yoginlily@126.com (Y.L.); 2 Hebei Agricultural Characteristic Industry Technical Guidance Station, Shijiazhuang 050021, China; 3 Shijiazhuang Seed Management Station, Shijiazhuang 050021, China

**Keywords:** actinobacteria, bacteria, *Bacillus subtilis*, fungi, *Trichoderma harzianum*

## Abstract

To study the effects of different microbial agents on the microbial community structure of continuously cropped strawberry soil after soil fumigation, seven treatments were applied: T1 (*Trichoderma harzianum* + *Bacillus subtilis* + *actinomycetes*), T2 (*Trichoderma harzianum* + *Bacillus subtilis*), T3 (*Trichoderma harzianum* + *actinomycetes*), T4 (CK) (water control), T5 (*Bacillus subtilis*), T6 (*actinomycetes*) and T7 (*Trichoderma harzianum*). A high-throughput sequencing platform (Illumina HiSeq 2500) was used to analyze the soil bacterial and fungal communities and their compositions. Compared with the T4 (CK) treatment, the application of microbial agents increased the richness and diversity of soil bacteria and fungi, and the effects of single microbial agents and compound microbial agents differed. The richness, diversity indices and population sizes of bacteria and fungi in the T6 treatment were the highest. The Chao1, observed species and Shannon indices of bacteria were 22.51%, 23.56% and 5.61% greater, respectively, than those of T4 (CK). The Chao1, observed species, Shannon and Simpson indices of fungi were 41.28%, 41.83%, 128.02% and 88.65% higher, respectively, than those of T4 (CK). At the genus level, the bacterial community compositions of T2 and T6 were the most similar, and the fungal community compositions of T1 and T5 were the most similar. Analysis of the genera in the dominant communities revealed that the application of microbial agents after dazomet fumigation increased the numbers and recovery rates of soil bacteria and fungi, especially the beneficial fungal genera, *Lecanicillium*, *Cladosporium*, *Saccharomyces* and *Aspergillus*. An investigation of strawberry growth and yield-related indicators revealed that the T6 treatment resulted in the lowest seedling mortality and the highest yield. In summary, adding microbial agents to soil with continuous cropping of strawberry after fumigation with dazomet is a scientifically sound and effective method for reconstructing the balance of the soil microbial flora and overcoming the obstacles associated with continuous cropping. In this study, the T6 (*actinomycetes*) treatment presented the best performance.

## 1. Introduction

Strawberry (*Fragaria × ananassa* Duch.) is a perennial herbaceous plant of the Rosaceae genus Fragaria and is an important horticultural crop worldwide [1]. China is the world’s largest strawberry producer and the largest strawberry consumer. According to FAO statistics, from 2018 to 2020, China’s strawberry production levels were 3.0691 million, 3.2082 million and 3.3367 million tons, respectively, ranking first in the world and exceeding those the second-ranked United States by 1.8852 million, 2.1731 million and 2.2807 million tons, respectively. However, owing to the limited number of cultivated lands in the strawberry planting areas in China, continuous cropping of strawberry is very common. Continuous cropping can lead to poor growth and development of strawberry plants, increase the incidence of soil-borne diseases, decrease yield and quality, and cause considerable losses in strawberry production. Among these factors, soil-borne diseases are a major problem in strawberry cultivation [2]. At present, the main methods used to control soil-borne diseases include soil fumigation, microbial agent-mediated control and organic fertilizer-mediated control [3].

Soil chemical fumigation is a commonly used method to control soil-borne diseases in the production of strawberry and other crops [4,5]. With the in-depth implementation of green, safe and environmental protection concepts, chloropicrin and bromomethane have been gradually eliminated in production because of their high toxicity and complicated operation. As a high-efficiency, low-toxicity, broad-spectrum comprehensive soil fumigation disinfectant with no residue, dazomet has a short retention time in the soil and is environmentally friendly. It can effectively decrease or kill most pathogenic bacteria in the soil and effectively prevent and treat soil-borne diseases [6]; in particular, its ability to inhibit Fusarium wilt is very obvious. Therefore, it is widely used in strawberry [7], tomato [8], cucumber [9] and other crops. However, the use of dazomet not only prevented the soil-borne diseases caused by continuous cropping but also changed the state of the soil environment. After fumigation with dazomet, the diversities and populations of pathogenic microorganisms and beneficial microorganisms in the soil are reduced on a large scale [10]. Parthipan et al. [11] reported that after dazomet fumigation, the number of microorganisms in the soil decreased significantly, the total number of bacteria decreased by 50%, and this effect lasted for at least 15 weeks.

Microbial agents can directly or indirectly improve the soil, maintain the balance of crop rhizosphere microflora, degrade toxic and harmful substances, play a role in controlling soil-borne diseases, enhance the growth of horticultural crops such as strawberries and tomatoes, increase fruit vitamin C contents and increase fruit commodity rates [12,13,14,15,16]. Studies have shown that the addition of beneficial bacteria after fumigation has little competition with other organisms and that they can easily re-establish and reproduce in the soil. In recent years, many studies have found that the combination of soil fumigation and microbial agents can help overcome the continuous cropping obstacle; that is, after chemical fumigation of soil, microbial agents are applied to the soil. This model not only overcomes the disadvantage of killing beneficial and harmful soil microorganisms together after the chemical fumigation of soil, but also solves the problem of the long time that it takes to overcome the continuous cropping obstacle by using microbial agents alone. For example, Wu et al. [17] reported that the addition of Trichoderma after soil fumigation effectively optimized soil ecological functions and increased crop yield. Cheng et al. [18] reported that adding biological fertilizers (e.g., Bacillus subtilis and Trichoderma harzianum) after the addition of soil fumigants effectively improved the soil and reshaped the soil microbial community structure. Li et al. [19] reported that the combination of dazomet fumigation and biological fertilizer (Bacillus) application effectively prevented the occurrence of soil-borne diseases and significantly promoted the growth of strawberry plants.

Many studies have investigated the use of dazomet fumigation or the sole application of microbial agents to alleviate the obstacles associated with continuous cropping in crop production. However, research on the control effects of additions of different microbial agents to continuously cropped strawberry soil after dazomet fumigation is still relatively scarce. The research team, following the previous production practice, determined that an amount of dazomet at 20 kg·667 m^−2^ can play a better role in prevention, control and promotion, and that this is more meaningful to save costs, increase efficiency and protect the environment. Therefore, in this study, dazomet fumigation (20 kg·667 m^−2^) was combined with different concentrations of microbial agents and compound microbial agents to treat soil used for continuous strawberry cultivation. The soil microbial communities and compositions were analyzed by a high-throughput sequencing platform, and the relationships between changes in the soil microbial communities and compositions and continuous cropping-related obstacles are discussed. This work clarifies the effects of adding microbial agents after dazomet fumigation on the diversity of the microbial populations of soils growing strawberry and provides a scientific basis for overcoming the obstacles associated with the continuous cropping of strawberry.

## 2. Materials and Methods

### 2.1. Experimental Materials

The *Trichoderma harzianum* chlamydospore agent used in the experiment was produced by Hainan Jinyufeng Bioengineering Co., Ltd., China, with an effective viable count of ≥2.0 billion CFU·g^−1^; the *Bacillus subtilis* culture was provided by the Institute of Plant Protection, Hebei Academy of Agriculture and Forestry Sciences, Shijiazhuang, China, with a concentration of 30 billion CFU·mL^−1^ (liquid formulation); and the actinomycetes were produced by Hebei Liangfu Biotechnology Co., Ltd., Shijiazhuang, China, with an effective viable count of ≥200 billion CFU·g^−1^. The tested strawberry variety was the main local variety, ‘Hongyan’.

### 2.2. Experimental Design

The experiment was carried out in the No. 15 greenhouse of the Junlebao Ecological Fruit and Vegetable Garden in Luquan District, Shijiazhuang City, China. The test soil was sandy loam, and strawberry plants were cultivated for 3 consecutive years. The last strawberry residue was removed, 2000 kg of organic fertilizer (cow dung compost) was applied as the base fertilizer, the soil was tilled to a depth of 15 cm, the straw was straightened and raked, and the soil was irrigated so that the soil water content reached 60%. The cotton top (20 kg·667 m^−2^) was evenly spread into the soil, and the soil was mixed with the medicine, fertilizer and soil. The soil was immediately covered with a polyethylene primary film with a thickness of 0.08 mm or more, and the soil was compacted around the film via the reverse pressure method (by furrowing and pressing). The film was disinfected in a closed greenhouse for 30 days. After fumigation, the vent of the greenhouse was opened first, the film was removed on the next day, the soil was tilled again, and the greenhouse was ventilated for 10 days. A high ridge from east to west had a height of 40 cm, a width of 50 cm and a distance of 40 cm. Two rows of strawberry plants were planted in each row, the plant spacing was 18 cm and the density was 8000 plants 667 m^−2^. The soil physical and chemical properties are shown in Table 1.

A single-factor randomized block design was used in the experiment, and a total of 7 treatments (dosage per 667 m^−2^) were set up as follows: T1, *Trichoderma harzianum* 800 g + *Bacillus subtilis* 2000 g + *Actinomycetes* 2000 g + brown sugar 200 g; T2, *Trichoderma harzianum* 800 g + *Bacillus subtilis* 2000 g; T3, *Trichoderma harzianum* 800 g + *actinomycetes* 2000 g + brown sugar 200 g; T4 (CK), water control; T5, *Bacillus subtilis* 4000 g; T6, *actinomycetes* 3000 g + brown sugar 200 g; and T7, *Trichoderma harzianum* 1000 g. Each treatment was repeated three times, and the plot area was 66 m^−2^, with 800 plants per plot (10 ridges). The microbial agents for each treatment were mixed with clear water to form a mixture. After planting, agent solutions were applied with a quantitative shoulder-back sprayer at 200 mL per hole. The cultivation and management techniques used were the same as those used for local production. Planting occurred in middle and late August, harvest occurred in early January of the next year, pulled into seedlings in early May of the next year, and irrigated with drip irrigation under the film.

### 2.3. Experimental Methods

Upon the maturation of the first fruit, the rhizosphere soils of the strawberry plants were sampled. Three soil samples were taken from each treatment via the three-point sampling method, fully mixed and stored at low temperature (dry ice). On February 24, the yields were measured. Three points were randomly selected for each treatment, and 100 plants were continuously investigated. The number of dead seedlings was determined, the average value was determined, and the percentage of dead seedlings was calculated. Thirty plants were selected at each point, the number of fruits per plant was counted, the average number of fruits per plant was calculated, and the average single-fruit weight was determined. The theoretical yield = fruit number per plant × fruit weight × plant number per plant × 0.85.

The methods for determining the physical and chemical properties of the soil were described by Wu et al. [17]. The soil genomic DNA was extracted with a ‘Omega Bio-tek’ Soil DNA Kit produced by Omega Company in the Irving, TX, USA. After gel electrophoresis and NanoDrop testing, the quality of each sample was determined. The Illumina MiSeq platform (China Shanghai Pasenuo Biotechnology Co., Ltd., Shanghai, China) was used to perform high-throughput sequencing of soil fungal ribosomal genes. The bacterial 16S-V3V4 (F, 5′-ACTCCTACGGGAGGCAGCA-3′; R, 5′-GGACTACHVGGGTWTCTAAT-3′) and fungal ITS-V1 (5′-ACTCCTACGGGAGGCAGCA-3′; R, 5′-GGACTACHVGGGTWTCTAAT-3′) primers were synthesized for PCR amplification. The bacterial 16S rRNA and fungal ITS gene sequences were aligned using the Silva database (https://www.arb-silva.de/, accessed on 16 September 2024).

### 2.4. Statistical Analysis

The DNA fragments of the community were sequenced via the Illumina platform, and the amplified sequence variants (ASVs) were generated after quality control, primer removal, denoising, splicing and chimera removal via the DADA2 method [20]. Microsoft Excel was used to organize the data. Alpha diversity (Chao1, observed species, Shannon and Simpson indices) was analyzed via QIIME2 (2019.4), the ggplot2 package (3.5.1) in R software (R 4.4.3) and SPSS 27.0 [21,22]. The VennDiagram package in R was used to construct a Venn diagram (https://www.genescloud.cn/chart/VennPlot, accessed on 16 September 2024). The heatmap in R was used to draw a heatmap of the species composition. QIIME2 (2019.4) and other software were used to compare and analyze the abundance of taxa at the phylum and genus levels in different soil treatments. SPSS 27.0 and Excel were used to analyze the growth and yield of the strawberry plants.

## 3. Results

### 3.1. Evaluation of the Sequencing Depth of the Soil Samples

**Figure 1:**
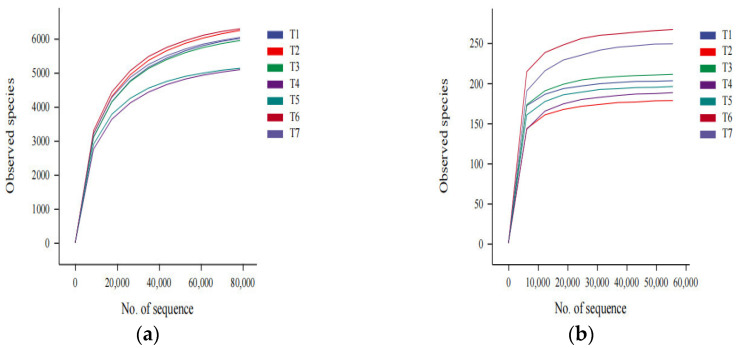
Bacterial (**a**) and fungal (**b**) sequencing dilution curves for the soil samples subjected to different treatments. Note: T1: *Trichoderma harzianum* + *Bacillus subtilis* + actinomycetes, T2: *Trichoderma harzianum* + *Bacillus subtilis*, T3: *Trichoderma harzianum* + actinomycetes, T4 (CK): water control, T5: *Bacillus subtilis*, T6: actinomycetes, and T7: *Trichoderma harzianum*. The following figures are the same representation.

A total of 2,799,194 pairs of reads were obtained by bacterial sequencing of the samples from seven soil treatments. After 100% similarity clustering, splicing and dechimerization, 2,215,822 high-quality sequences were obtained after paired-end read splicing and filtering, and 35,965 ASVs were obtained by clustering. A total of 1,958,850 pairs of reads were obtained by fungal sequencing. Clustering, splicing and dechimerization were performed with 100% similarity. After splicing and filtering, 1,711,313 high-quality sequences were obtained. A total of 1447 ASVs were obtained by clustering. The sequencing reads were randomly selected, and dilution curves were constructed according to the number of sequences extracted and the number of species they could represent. Figure 1 shows that the curves for bacteria and fungi tended to gradually flatten, indicating that the sequencing data were reliable.

### 3.2. Soil Bacterial and Fungal Community Richness and Diversity Analysis

We used SPSS 27.0 to analyze the Chao1 index, species number, Shannon index and Simpson index of the different soil treatments. The Chao1 estimator and observed species indices were used to reflect the species richness of the communities, and the Shannon and Simpson indices were used to reflect the species diversities. The richness and diversity indices of the bacterial communities in the different soil treatments were analyzed. Table 2 shows that the richness indices of the soils in the treatments with microbial agents were significantly greater than those in the T4 treatment (CK), except for those in the T5 treatment. In the treatments with microbial agents, the Chao1 and observed species indices of T6 were the highest, with values 22.51% and 23.56% greater than those of T4 (CK), respectively. The Simpson index for T4 (CK) was significantly lower than those of the other treatments. The Shannon and Simpson indices of T6 were the highest, and the Shannon index of T6 was significantly greater than those of the other treatments and 5.61% greater than that of T4 (CK).

The richness indices of T6 were significantly greater than those of T1, T2, T3, T4 (CK) and T5, and the Chao1 index and observed species index were 41.28% and 41.83% greater, respectively, than those for T4 (CK). The diversity indices of T4 (CK) were significantly lower than those of the other treatments. The Shannon indices of T1, T2, T3, T5, T6 and T7 were 105.45%, 36.58%, 83.27%, 56.42%, 128.02% and 87.55% greater than that of T4 (CK), respectively. The Simpson indices of T1, T2, T3, T5, T6 and T7 were 82.92%, 40.23%, 76.79%, 53.34%, 88.65% and 67.05% greater than that of T4 (CK), respectively, with T6 exhibiting the greatest increase. Compared with T4 (CK), the application of microbial agents increased the richness and diversity of soil bacteria and fungi after the soil was subjected to dazomet fumigation, and the increases observed in single microbial agents and compound microbial agents were different. The richness and diversity indices of bacteria and fungi in the T6 treatment were the highest.

### 3.3. Analysis of the Soil Bacterial and Fungal Groups

The number of bacterial ASVs in each sample was determined with a similarity threshold of 100%. As shown in Table 1, T4 (CK) had the lowest number (10,856) of ASVs, and T6 had the most ASVs, 31.42% greater than that of T4 (CK). The numbers of ASVs in T2, T3, T7, T1 and T5 were 28.69%, 21.86%, 21.01%, 20.00% and 3.52% greater, respectively, than that in T4 (CK). Analysis of the number of ASVs in fungal samples revealed that T2 had the lowest number of ASVs (356), which was 8.95% lower than that of T4 (CK). The other treatments resulted in increased numbers of ASVs; the number of ASVs in T6 increased the most, reaching 616, which was 57.54% greater than that in T4 (CK). The numbers of ASVs in T7, T3, T1 and T5 were 42.20%, 14.83%, 10.49% and 5.63% greater than that in T4 (CK), respectively.

**Figure 2:**
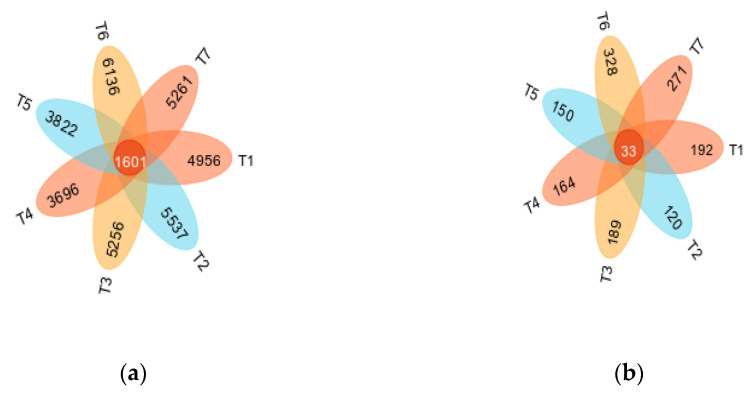
Venn diagrams of soil bacteria (**a**) and fungi (**b**) under different treatments.

The Venn diagrams reflect the number of common and unique ASVs between different samples and intuitively show the overlap of ASVs between samples. According to the Venn diagrams of the bacterial and fungal ASV distributions (Figure 2), the number of ASVs shared by bacteria was 1601 in the rhizosphere soil samples from the seven treatments at 100% similarity. The numbers of ASVs specific to T1, T2, T3, T4 (CK), T5, T6 and T7 were 4956, 5537, 5256, 3696, 3822, 6136 and 5261, respectively, accounting for 45.65%, 39.63%, 39.73%, 28.37%, 34.01%, 43.01% and 40.05%, respectively, of the total number of ASVs in each sample. The number of ASVs shared by fungi was 33. The number of ASVs specific to T1, T2, T3, T4 (CK), T5, T6 and T7 were 192, 120,189, 164, 150, 328 and 271, respectively, accounting for 44.44%, 33.71%, 42.09%, 41.94%, 36.32%, 53.25% and 48.74%, respectively, of the total ASVs of each sample. The results revealed that the soil bacterial and fungal communities under treatments with different microbial agents presented certain differences. The number of bacterial ASVs in the soils treated with microbial agents was greater than that in the CK treatment, and the number of fungal ASVs was greater than that in the CK treatment, except in the T2 treatment (*Trichoderma harzianum* + *Bacillus subtilis*).

### 3.4. Analysis of the Soil Bacterial and Fungal Community Compositions

#### 3.4.1. Analysis of the Soil Bacterial and Fungal Phylum-Level Community Compositions

**Figure 3:**
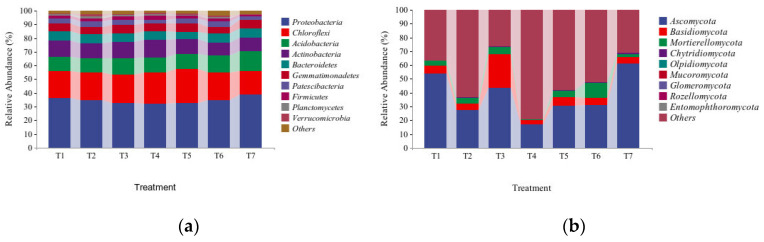
Analysis of the species compositions of the soil bacterial (**a**) and fungal (**b**) communities at the phylum level under different treatments.

Figure 3a shows that the bacteria detected in the strawberry soil under the seven different treatments were mainly from four phyla, namely, *Proteobacteria*, *Chloroflexi*, *Acidobacteria* and *Actinobacteria*. Among them, *Proteobacteria* accounted for the greatest proportions, reaching 36.43% (T1), 34.94% (T2), 32.81% (T3), 32.32% (T4), 32.50% (T5), 34.85% (T6), and 38.91% (T7) in the seven soil treatments. Compared with T4 (CK), the relative abundances of *Proteobacteria* in the T1, T2, T3, T5, T6 and T7 soils increased, whereas the relative abundances of *Actinobacteria* decreased. In T1, T2, T3, T5, T6 and T7, the abundances of *Proteobacteria* increased by 12.7%, 9.2%, 2.55%, 1.56%, 8.9% and 21.61%, respectively, whereas the abundances of *Actinobacteria* decreased by 11.5%, 17.28%, 8%, 18.21%, 31.41% and 23.98%, respectively.

As shown in Figure 3b, the fungi detected in the strawberry soils under the seven different treatments were associated with three main phyla, namely, *Ascomycota*, *Basidiomycota* and *Mortierellomycota*. The relative abundances of *Ascomycota* were the highest in all the soil treatments, reaching 53.87% (T1), 27.2% (T2), 43.63% (T3), 17.26% (T4), 30.61% (T5), 30.97% (T6) and 61.33% (T7) in the seven soil treatments. Compared with those in the T4 treatment (CK), the relative abundances of *Ascomycota*, *Basidiomycota* and *Mortierellomycota* in the T1, T2, T3, T5, T6 and T7 treatments increased. In T1, T2, T3, T5, T6 and T7, the abundances of *Ascomycota* increased by 36.61%, 9.95%, 26.38%, 13.36%, 13.71% and 44.07%, respectively; the abundances of *Basidiomycota* increased by 2.71%, 2.06%, 21.58%, 3.21%, 2.16% and 1.37%, respectively; and the abundances of *Mortierellomycota* increased by 3.27%, 3.74%, 4.27%, 4.39%, 10.77% and 1.70%, respectively. The results showed that the addition of microbial agents after dazomet fumigation affected the proportions of bacteria and fungi.

#### 3.4.2. Analysis of the Soil Bacterial and Fungal Community Compositions at the Genus Level

**Figure 4:**
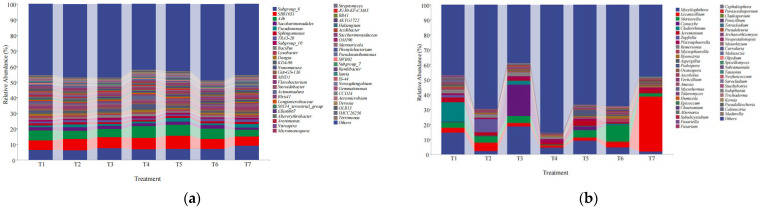
Analysis of the species compositions of the soil bacterial (**a**) and fungal (**b**) communities at the genus level under different treatments.

**Figure 5:**
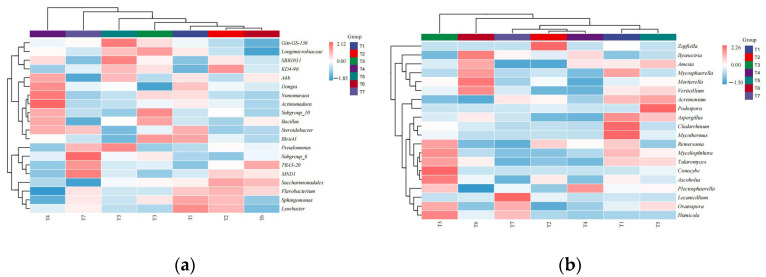
Cluster analysis of bacteria (**a**) and fungi (**b**) in different soil treatments at the genus level.

The species that were identified at the genus level and ranked in the top 50 in terms of relative abundance were analyzed, and the remaining species, with their relative abundances combined, were classified as “other.” The compositions of the soil fungal communities in the different treatment samples also changed significantly (Figure 4). The soil bacteria and fungi in each treatment were mainly concentrated in 20 genera in terms of abundance, so a heatmap of the top 20 genera was drawn, and cluster analysis was carried out. As shown in Figure 5a, T1 presented a significantly greater abundance of one genus (*Lysobacter*), T2 presented a significantly greater abundance of one genus (*KD4-96*), T3 presented a significantly greater abundance of one genus (*Subgroup_10*), and T4 presented significantly greater abundances of three genera (*Actinomadura, Nonomuraea* and *Dongia*) than the other treatments. Compared with those of the other treatments, the bacterial community of T5 presented significantly greater abundances of three genera (*Gitt-GS-136*, *SBR1031* and *Pseudomonas*). Compared with those of the other treatments, the fungal community of T7 presented significantly greater abundances of two genera (*Subgroup_6* and *MND1*). The bacterial community compositions in the different treatment samples could be divided into six categories: the T2 and T6 community compositions were similar and clustered in the first category; the community compositions of T1, T3, T4, T5 and T7 were quite different from those of the other treatments, and these were clustered in the second to sixth categories.

As shown in Figure 5b, two fungal genera, *Cladorrhinum* and *Mycothermus*, were significantly more abundant in the T1 treatment than in the other treatments. The fungal community of T2 presented a significantly greater abundance of one genus (*Zopfiella*) than did the other treatments; the fungal community of T3 presented a significantly greater abundance of one genus (*Conocybe*) than did the other treatments; the fungal community of T5 presented a significantly greater abundance of one genus (*Podospora*) than did the other treatments; the fungal community of T6 presented significantly greater abundances of two genera (*Ilyonectria* and *Mortierella*) than did the other treatments; and the fungal community of T7 presented a significantly greater abundance of one genus (*Lecanicillium*) than did the other treatments. The fungal community compositions in the soil samples from the different treatments could be divided into four categories: T1 and T5 were clustered into the first category; T2, T4 and T7 were clustered into the second category; T6 was clustered into the third category; and T3 was clustered into the fourth category. The compositions of the T1 and T5 communities were similar, and the compositions of the T2, T4 and T7 communities were similar, whereas the compositions of the T6 and T3 communities were quite different from those of the other treatments, with each representing a class.

#### 3.4.3. Analysis of the Dominant Soil Bacterial and Fungal Communities at the Genus Level

A relative abundance > 1% was used as the standard for the dominant flora. According to Table 3, there were 9, 9, 9, 11, 9, 8 and 9 dominant bacterial genera in the T1–T7 treatments, accounting for 27.56%, 27.98%, 28.67%, 32.07%, 31.43%, 27.63% and 28.21% of the communities, respectively. There were six common dominant bacterial communities in the seven treatment groups: *Subgroup_6*, *SBR1031*, *A4b*, *Saccharimonadales*, *Pseudomonas* and *Sphingomonas*.

There were 11, 6, 7, 5, 8, 5 and 5 dominant fungal genera in the T1–T7 treatments, accounting for 47.51%, 25.11%, 54.13%, 10.89%, 27.87%, 23.32% and 44.66% of the communities, respectively. There were two common dominant fungal communities in the seven treatments: *Myceliophthora* and *Lecanicillium*. In addition to the common dominant fungal groups, compared with those in the control (T4), the dominant genera in T1 included *Mortierella*, *Cladorrhinum*, *Zopfiella*, *Mycosphaerella*, *Aspergillus* and *Mycothermus*. T2 also included *Mortierella* and *Zopfiella*. T3 also included *Mortierella*, *Conocybe* and *Cladorrhinum*. T5 also included *Mortierella*, *Conocybe*, *Aspergillus* and *Zopfiella*. T6 also included *Mortierella*, *Ilyonectria* and *Mycosphaerella*. T7 also included *Mortierella*. The beneficial fungi in the dominant flora of each treatment also differed. T1 contained four beneficial fungal genera, *Lecanicillium*, *Cladorrhinum*, *Remersonia* and *Aspergillus*; T2 contained two beneficial fungal genera, *Lecanicillium* and *Remersonia*; T3 contained three beneficial fungal genera, *Lecanicillium*, *Cladorrhinum* and *Remersonia*; T4 contained two beneficial fungal genera, *Lecanicillium* and *Remersonia*; T5 contained two beneficial fungal genera, *Lecanicillium* and *Aspergillus*; T6 contained two beneficial fungal genera, *Lecanicillium* and *Cladorrhinum*; and T7 contained one beneficial fungal genus, *Lecanicillium*.

### 3.5. Effects of Different Treatments on the Growth and Yield of Strawberry

We used SPSS 27.0 to analyze the seedling mortalities, numbers of surviving plants, average numbers of fruits per plant, average single-fruit weights, and yields of the different soil treatments. Table 4 shows that after the soil was fumigated with dazomet, the effects of adding microbial agents on seedling mortality, the number of surviving plants and the yield of strawberry differed. There was no significant difference in the percentages of dead seedlings between T4 (CK) and T2, but there were significant differences between T4 (CK) and the other treatments. The yield per unit area of T4 (CK) was significantly lower than those of T3, T6 and T7. The seedling mortality of T6 was the lowest, significantly lower than that of T4, and the yield per unit area of T6 was the highest, significantly greater than those of T2, T4 and T5.

## 4. Discussion

In healthy soil ecosystems, beneficial and harmful microorganisms exist in a dynamic balance in terms of type and quantity [23,24]. Continuous cropping leads to the enrichment of certain microorganisms, increases the abundance of soil-borne pathogens, and reduces the diversity and abundance of beneficial bacteria, resulting in an imbalance in soil microbial populations. An imbalance in the soil microbial population structure can reduce crop yield and even lead to crop failure [4,25]. Therefore, it is important to study the obstacles associated with the continuous cropping of strawberry plants to reasonably and effectively inhibit the growth of soil-borne pathogens and reconstruct healthy soil microflora.

At present, in strawberry cultivation, dazomet and other agents are usually used to fumigate the soil to kill the vast majority of pathogenic bacteria; this treatment also greatly impacts the beneficial groups in the soil [7,26]. The application of microbial agents is an effective method to increase the abundances of beneficial bacteria and fungi in soils and improve the soil microbial population structure [27]. This study revealed that, compared with T4 (CK), the application of microbial agents after dazomet fumigation increased the richness and diversity of the soil bacterial and fungal communities, which is consistent with the results of Li et al. [7], and the effects of applying single microbial agents were different from those of applying compound microbial agents. The richness, diversity indices and population sizes of bacteria and fungi in the T6 treatment were the highest. Compared with those in the T4 treatment (CK), the relative abundances of *Proteobacteria* in the T1, T2, T3, T5, T6 and T7 soil treatments increased by 12.7%, 9.2%, 2.55%, 1.56%, 8.9% and 21.61%, respectively. *Proteobacteria* play important roles in ecological processes such as the nitrogen cycle and organic matter decomposition [28]. At the fungal phylum level, compared with those in T4 (CK), the relative abundances of *Ascomycota*, *Basidiomycota* and *Mortierellomycota* in the T1, T2, T3, T5, T6 and T7 soils increased, with the relative abundances of *Ascomycota* increasing by 36.61%, 9.95%, 26.38%, 13.36%, 13.71% and 44.07%, respectively. *Ascomycota* play important roles in soil microbial decomposition, soil nutrient cycling and ecosystem stability [29,30]. These findings indicate that the application of microbial agents affects the growth and reproduction of some rhizosphere microorganisms and changes the bacterial and fungal community structures.

Compared with those in the treatments with microbial agents, there were only five dominant fungal genera with abundances greater than 1% in T4 (CK): *Myceliophthora* (4.27%), *Plectosphaerella* (2.55%), *Lecanicillium* (1.50%), *Remersonia* (1.23%) and *Acremonium* (1.23%). The abundances of other fungi were low, and some fungal populations did not recover. In addition, this study revealed that the application of microbial agents, especially *Lecanicillium*, *Cladorrhinum*, *Remersonia* and *Aspergillus*, to soils used for continuous cropping of strawberry after dazomet fumigation significantly increased the abundances and recovery rates of the soil bacterial and fungal populations. The recovery rates of beneficial fungi were significantly accelerated. *Lecanicillium* has been developed as a biological insecticide and is widely used in agricultural production for the biological control of insects [31]. *Cladorrhinum* can not only promote plant growth but also control plant pathogens [32]. *Remersonia* inhibits the development of pathogens and improves soil properties [33]. *Aspergillus* can purify soil contaminated by heavy metals, oil spills and microbial toxins [34]. When microbial agents were added in this study, the recovery rate of *Lecanicillium* was the fastest, and the abundances in each treatment exceeded that in the T4 (CK) treatment, with the abundance in T7 (*Trichoderma harzianum*) reaching 36.5%. The abundance in T1 (*Trichoderma harzianum* + *Bacillus subtilis* + *actinomycetes*) reached 12.81%. The abundances of *Remersonia* and *Aspergillus* were also high. In T7, the abundances of beneficial fungi were relatively low (0.00%~0.47%), except for the abundance of *Lepidotheca*, which was the highest (36.55%). The abundances of other fungi were low, with the abundances of *Trichoderma*, *Cladosporium*, *Remersonia*, *Chaetomium* and other fungi being 0.00%. The reason for this phenomenon may be that after the soil used for continuous cropping of strawberry is fumigated with dazomet, microbial agents, especially beneficial bacteria and fungi, are added to enrich the soil bacterial and fungal communities. When beneficial bacteria and fungi occupy the vacant niche, the soil bacteria and fungi tend to compensate dynamically for the increased abundances of beneficial bacterial and fungal species, reducing seedling mortality and increasing yield. Among the treatments, the T6 (*actinomycetes*) treatment resulted in the lowest seedling mortality and the highest yield. The T2 and T4 treatments resulted in the greatest seedling mortalities and lowest yields. Compared with previous studies, this experiment is no longer limited to the application of a certain microbial inoculant alone, but on the basis of the application of microbial agents alone, a compound microbial agent is added, and the corresponding concentration is set to find the best treatment to maximize crop yield. Moreover, the soil microbial community can be effectively reconstructed to prevent soil-borne diseases.

## 5. Conclusions

In summary, after the soils used for continuous cropping of strawberry were fumigated with dazomet, microbial agents, especially beneficial bacteria and fungi, were added to enrich the soil bacterial and fungal communities. When beneficial bacteria and fungi occupy the vacant niche, the soil flora tend to compensate dynamically for the increased abundances of beneficial bacterial and fungal species, reducing the rate of seedling mortality and increasing yield. Among them, a single application of *actinomycetes* resulted in the lowest seedling mortality and the highest yield. The application of *Trichoderma harzianum* + *Bacillus subtilis* as a microbial agent and the water control resulted in the highest seedling mortalities and lowest yields. In addition to reasonably and effectively inhibiting soil-borne pathogens, reconstructing the microbial flora of soil through microbial agents is a scientific and effective method to alleviate continuous cropping obstacles, and the application of *actinomycetes* alone is best. However, although the experimental samples were repeated three times, only the samples of one year were tested. Therefore, the role of microbial agents in the prevention and treatment of strawberry soil continuous cropping obstacles needs to be further verified by later experiments. In addition, in follow-up work, the soil microbial community associated with dazomet fumigation combined with microbial agents should be further dynamically tracked, and the occurrence of soil-borne diseases and changes in soil environmental factors should be dynamically monitored to clarify the mechanism by which microbial agents are applied to promote strawberry growth after fumigation.

## Figures and Tables

**Table 1:** Soil physicochemical properties.

Alkali-Hydrolyzed Nitrogen (mg·kg^−1^)	Available Phosphorus (mg·kg^−1^)	Available Potassium (mg·kg^−1^)	Organic Matter (g·kg^−1^)	pH	Conductivity (μs/cm)
133.47	212.27	386.33	22.9	7.0	302.5

**Table 2:** Abundance and diversity indices of the soil fungal communities under different treatments.

Kingdom	Treatment	ASVs	Community Abundance Index		Community Diversity Index	
			Chao1 Index	Observed Species	Shannon	Simpson
			*p* < 0.05			
Bacteria	T1	13,027	6335.19 ± 250.41 a	6008.70 ± 233.75 a	11.26 ± 0.17 ab	0.9988 ± 0.0005 a
	T2	13,971	6551.68 ± 273.69 a	6242.50 ± 239.18 a	11.35 ± 0.28 ab	0.9988 ± 0.0005 a
	T3	13,229	6236.40 ± 121.48 a	5944.23 ± 69.11 a	11.27 ± 0.60 ab	0.9988 ± 0.0002 a
	T4 (CK)	10,856	5332.01 ± 210.10 b	5089.53 ± 156.99 b	10.87 ± 0.01 b	0.9981 ± 0.0001 b
	T5	11,238	5308.26 ± 289.95 b	5129.43 ± 222.82 b	11.12 ± 0.21 ab	0.9987 ± 0.0002 a
	T6	14,267	6532.83 ± 504.53 a	6288.47 ± 445.66 a	11.48 ± 0.11 a	0.9991 ± 0.0001 a
	T7	13,137	6332.29 ± 417.81 a	6036.10 ± 206.82 a	11.40 ± 0.14 ab	0.9990 ± 0.0002 a
Fungi	T1	432	203.90 ± 7.86 bc	203.13 ± 8.33 bc	5.28 ± 0.31 ab	0.9298 ± 0.0238 ab
	T2	356	179.95 ± 8.52 c	178.50 ± 7.88 c	3.51 ± 0.22 d	0.7128 ± 0.0184 d
	T3	449	212.27 ± 9.38 b	210.90 ± 15.68 b	4.71 ± 0.16 bc	0.8986 ± 0.0254 ab
	T4 (CK)	391	189.84 ± 10.65 bc	187.97 ± 15.22 bc	2.57 ± 0.41 e	0.5083 ± 0.0694 e
	T5	413	196.55 ± 22.28 bc	195.77 ± 21.78 bc	4.02 ± 0.32 cd	0.7794 ± 0.0758 cd
	T6	616	268.21 ± 13.31 a	266.60 ± 12.52 a	5.86 ± 0.70 a	0.9589 ± 0.0211 a
	T7	556	250.93 ± 20.05 a	249.23 ± 20.58 a	4.82 ± 0.90 bc	0.8491 ± 0.0457 c

Note: means followed by different letters are significantly different at *p* < 0.05.

**Table 3:** Changes in the abundances of the dominant bacteria and fungi in the soil under the different treatments.

Kingdom	Serial Number	Genus	Relative Abundance (%)						
			T1	T2	T3	T4	T5	T6	T7
	1	*Subgroup_6*	6.48	6.25	7.48	6.96	7.10	7.07	9.15
	2	*SBR1031*	5.98	7.15	7.01	7.29	8.31	6.49	5.93
	3	*A4b*	6.52	5.41	5.37	7.45	7.18	6.44	4.35
	4	*Saccharimonadales*	2.01	2.28	1.98	1.76	1.92	2.20	1.50
	5	*Pseudomonas*	1.19	1.55	1.05	1.02	2.37	1.44	2.04
	6	*Sphingomonas*	1.46	1.42	1.34	1.09	1.22	1.15	1.34
	7	*TRA3-20*	/	1.29	1.20	1.00	1.17	1.62	1.70
	8	*Subgroup_10*	1.04	1.17	1.58	1.46	/	/	1.01
	9	*Bacillus*	/	/	1.66	1.62	1.14	1.22	/
	10	*Lysobacter*	1.65	1.46	/	1.01	/	/	1.19
	11	*Dongia*	1.23	/	/	1.41	1.02	/	/
	The relative abundance > 1% of the genus number		9	9	9	11	9	8	9
	Dominant genus proportion/%		27.56	27.98	28.67	32.07	31.43	27.63	28.21
	1	*Myceliophthora*	14.64	2.17	18.81	4.27	9.17	4.67	1.96
	2	*Lecanicillium*	3.04	5.48	1.97	1.50	2.13	3.61	36.55
	3	*Mortierella*	3.86	4.34	4.87	/	4.99	11.37	2.30
	4	*Conocybe*	/	/	20.98	/	2.20	/	/
	5	*Cladorrhinum*	12.81	/	2.61	/	/	/	/
	6	*Acremonium*	3.29	2.23	/	1.23	3.70	/	2.50
	7	*Zopfiella*	1.52	8.85	/	/	/	/	/
	8	*Plectosphaerella*	1.39	/	2.06	2.55	1.53	/	1.35
	9	*Remersonia*	2.13	2.04	2.83	1.34	/	/	/
	10	*Mycosphaerella*	2.04	/	/	/	/	2.14	/
	11	*Ilyonectria*	/	/	/	/	/	1.53	/
	12	*Aspergillus*	1.53	/	/	/	1.13	/	/
	13	*Podospora*	/	/	/	/	3.02	/	/
	14	*Mycothermus*	1.26	/	/	/	/	/	/
	The relative abundance > 1% of the genus number		11	6	7	5	8	5	5
	Dominant genus proportion/%		47.51	25.11	54.13	10.89	27.87	23.32	44.66

**Table 4:** Effects of different treatments on the growth and yield of strawberry.

Treatment	Seedling Mortality (%)	Number of Surviving Plants (667 m^−2^)	Average Fruit per Plant (Units)	Average Single Fruit Weight (g^−1)^	Yield kg·667 m^−2^
	*p* < 0.05				
T1	4.7 ± 2.52 b	7626.7 ± 201.33 a	7.3 ± 0.15 a	24.3 ± 0.47 a	1143.4 ± 15.8 abcd
T2	10.7 ± 3.79 a	7146.7 ± 302.88 b	7.3 ± 0.25 a	24.4 ± 0.70 a	1088.2 ± 13.89 cd
T3	3.7 ± 1.53 b	7706.7 ± 122.2 a	7.4 ± 0.1 a	24.7 ± 0.42 a	1181.2 ± 14.73 ab
T4	12.3 ± 1.53 a	7013.3 ± 122.2 b	7.3 ± 0.46 a	24.5 ± 0.66 a	1065.5 ± 71.11 d
T5	6.3 ± 1.53 b	7493.3 ± 122.2 a	7.2 ± 0.36 a	24.0 ± 0.42 a	1102.5 ± 79.92 bcd
T6	2.3 ± 0.58 b	7813.3 ± 46.19 a	7.3 ± 0.21 a	24.6 ± 1.22 a	1195.5 ± 31.68 a
T7	4.3 ± 3.97 b	7653.3 ± 122.2 a	7.4 ± 0.12 a	24.0 ± 0.61 a	1152.0 ± 28.46 abc

Note: means followed by different letters are significantly different at *p* < 0.05.

## Data Availability

The original contributions presented in this study are included in the article. Further inquiries can be directed to the corresponding authors.
